# Unique gel-like colony forming bacterium *Novosphingobium pituita* sp. nov., isolated from a membrane bioreactor (MBR) treating sewage

**DOI:** 10.1016/j.heliyon.2024.e38795

**Published:** 2024-10-01

**Authors:** Tomoya Ikarashi, Uchini S. Bandaranayake, Takahiro Watari, Takashi Yamaguchi, Masashi Hatamoto

**Affiliations:** a Department of Civil and Environmental Engineering, Nagaoka University of Technology, Niigata, 940-2188, Japan; b Department of Science of Technology Innovation, Nagaoka University of Technology, Niigata, 940-2188, Japan

**Keywords:** Biofilm, Gelatinous colony, Extracellular polymeric substances, Extracellular polysaccharide

## Abstract

A novel, gelatinous, colony-forming, rod-shaped bacterial strain, designated IK01^T^ was isolated from biofilms formed on the membrane surface of a sewage-treating membrane bioreactor (MBR). Strain IK01^T^ produced gelatinous and almost transparent colonies at lower medium concentrations. Fourier transform infrared analysis of the gelatinous colony matrix showed that the matrix could be a biofilm substance. This suggests that strain IK01^T^ is a fouling-causing bacteria in the MBR. Furthermore, 16S rRNA gene sequence analysis showed that strain IK01^T^ was phylogenetically placed in the genus *Novosphingobium.* The average nucleotide identity values for IK01^T^ and the other 50 species of the genus *Novosphingobium* ranged from 78.5 to 83.9 %. Correspondingly, the estimated digital DNA-DNA hybridization values ranged from 20.8 to 24.4 %. The genomic DNA G + C content was 66.0 %. The predominant fatty acids were summed feature 8 (C_18:1_*ω*7*c* and/or C_18:1_*ω*6*c*), summed feature 3 (C_16:1_*ω*7*c* and/or C_16:1_*ω*6*c*), and C_14:0_ 2-OH. A polar lipid profile revealed phosphatidylethanolamine, two unidentified phospholipids, and three aminoglycophospholipids as major compounds. The major respiratory quinone was ubiquinone Q-10. Genotypic, chemotaxonomic, and phenotypic analyses characterized the newly identified strain IK01^T^, as a novel species of the genus *Novosphingobium,* for which we propose the name *Novosphingobium pituita* sp. nov. The type strain is IK01^T^ (NBRC 116408^T^ = DSM 116658^T^).

## 1 Introduction

*Novosphingobium* is a genus within the class Alphaproteobacteria [1] of the family *Sphingomonadaceae* or *Erythrobacteraceae*, which was proposed by Takeuchi et al. (2001) [2] by dividing the genus *Sphingomonas* based on phylogenetic and chemotaxonomic analyses. As of February 2024, over 60 species with validly published names in this genus according to the list of prokaryotic names with standing in nomenclature (LPSN; https://lpsn.dsmz.de).

Typically, the genus *Novosphingobium* comprises rod-shaped, small bacilli that are non-sporulating, aerobic or facultative anaerobic, gram-negative, and chemoorganotrophic bacteria [2,3]. Chemotaxonomic analysis has revealed that ubiquinone Q-10 is the predominant respiratory quinone in the genus, and spermidine is the predominant polyamine [2,4]. The major fatty acid in the genus are C18:1w7c and/or C 18:1w6c, and sphingoglycolipids are present in *Novosphingobium* species with a genomic G + C content of 62–71 % [2,5,6].

*Novosphingobium* is commonly isolated from freshwater, seawater, plants [5], soil, sediment [7], groundwater, rhizospheres, fish culture ponds, sewage ponds, activated sludge [6], wastewater treatment plants [8,9], coastal or freshwater sediments, lake surface water layers [10], and contaminated groundwater bioremediation reactors [11]. In addition, this genus produces extracellular polymeric substances (EPS) that are affected by environmental and culture conditions [12,13].

Membrane bioreactors (MBR) are widely used to effectively treat wastewater and can produce high-quality treated water [14,15]. However, this group of microorganisms forms a gel-like structure (biofilm) on the membrane surface, causing membrane fouling, which reduces the effectiveness of the reactor and becomes a crucial problem [16,17]. Therefore, the identification of biofilm-forming bacteria on surfaces is important for developing effective control measures [17,18]. During the identification of biofilm-forming bacteria in the MBR, the gelatinous-textured colony-forming bacterial strain IK01^T^ was isolated from a biofilm sample collected from the membrane surface, resulting in biofouling. In this study, we characterized a new strain, IK01^T^, of the genus *Novosphingobium* and proposed a novel species.

## 2 Materials and methods

### 2.1 Isolation and culture conditions

Gel-like biofilm samples were collected from the membrane surface of an experimental MBR at Nagaoka Central Sewage Treatment Center in Nagaoka, Japan [19]. When membrane fouling occurred, the fouled membrane was removed from the MBR, the membrane surface was rinsed with ultrapure water to remove loosely attached sludge, and the tightly bonded gel-like biofilm samples were collected by squeezing. The collected samples were stored at 4 °C until further use. The biofilm samples were diluted with ultrapure water, spread on 1 % R2A agar (1 % [w/v]) plates, and incubated at 28 °C for 2 weeks. The strain was purified by repeatedly streaking single colonies on normal R2A plates. Stock cultures were preserved at −80 °C in R2A medium supplemented with 10 % glycerol (v/v).

### 2.2 Phylogenetic analysis

Total genomic DNA was extracted using the FastDNA Spin Kit for Soil (MP Biomedicals), following the manufacturer's instructions. PCR amplification of the 16S rRNA gene was performed using Back 8F mix (5′-AGA GTT TGA TYM TGG CTC AG-3′) and Univ. 1492 rm (5′-GGGH TAC CTT GTT ACG ACT T-3′) [20]. The PCR product was purified using a QIAquick PCR Purification Kit (QIAGEN) and subjected to Sanger sequencing after confirming its quality by agarose gel electrophoresis. The nearly complete 16S rRNA gene sequence of strain IK01^T^ (1379 bp) was compared with available gene sequences of validly published species on the EzBioCloud server (https://www.ezbiocloud.net/) [21]. Phylogenetic trees based on the 16S rRNA sequences of strain IK01^T^ and related type strains of members of the genus *Novosphingobium* were reconstructed by neighbor-joining, maximum-likelihood, and maximum-parsimony methods using the MEGA 11 software package [22]. The robustness of the phylogenetic trees was evaluated using 1000 bootstrap resampling analyses [23].

For the whole-genome-based taxonomic analysis, the genome sequence data were analyzed using the Type Strain Genome Server (TYGS) [24].

### 2.3 Physiology and chemotaxonomy

After overnight incubation (28 °C) in R2A medium with shaking, cell morphology of strain IK01^T^ was observed using a phase-contrast light microscope (BX53, OLYMPUS). The motility of IK01^T^ was investigated by incubating the strain in R2A medium supplemented with soft agar (0.5 %, w/v) for 24 h at 28 °C. Changes in the colony characteristics of strain IK01^T^ were investigated by culturing it on varying concentrations of R2A medium and agar. The media concentrations were 1 %, 10 %, and 100 % along with 0.5 %, 1.0 %, and 1.5 % agar was added to each medium (all concentrations in w/v%) and incubated at 28 °C for 1 week. *Novosphingobium capsulatum* NBRC 12533^T^ was cultured under the same conditions as a reference. The optimal pH for the strain was determined by varying pH (4–10 in intervals of 1.0 pH units) with R2A medium using appropriate buffer systems: 0.5 M Tris-HCl (pH 8–10), 0.5 M MOPS (pH 7), 0.5M MES (pH 5–6), and 0.1 M citrate buffer (pH 4). The effects of temperature (4 °C, 10 °C, 15 °C, 20 °C, 28 °C, 37 °C, 46 °C, and 55 °C) on the growth of the strain on the R2A plate were examined. Salt tolerance of the strain was examined by growth on R2A medium supplemented with different salinity conditions (0.5–4.0 % [w/v] at intervals of 0.5 %). Substrate oxidation and biochemical tests were performed using API 20NE (bioMerieux Japan), following the manufacturer's instructions. Strains IK01^T^ and *N. capsulatum* NBRC 12533^T^ were incubated overnight (28 °C) in LB medium with shaking, then collected and washed with saline, the microbial densities adjusted to a McFarland standard of 0.5, and were subsequently subjected to the API 20NE test. Antibiotic sensitivity tests were performed on R2A plates using disc diffusion methods based on the Kirby-Bauer method [25,26]. Antibiotic discs containing 30 μg per disc of the following antibiotics: ampicillin, chloramphenicol, nalidixic acid, penicillin G, rifampicin, tetracycline, kanamycin, streptomycin, and vancomycin, were mounted on each plate and incubated at 28 °C for 2 days.

Fourier transform infrared (FTIR) analysis of the gelatinous colonies was conducted using the modified KBr pellet method. An FTIR spectrometer (FTIR 4100, JASCO Corporation) equipped with a diffusion-reflection measurement instrument was used.

For cellular fatty acid composition, respiratory quinone, and polar lipid analyses, we used strain IK01^T^ cultivated aerobically on R2A medium at 28 °C for 3 days with shaking. Fatty acids were extracted from freeze-dried cells according to the Sherlock Microbial Identification System protocol (Version 6.0, MIDI). The extracted fatty acids were analyzed using gas chromatography and identified using the TSBA6 library. For respiratory quinone analysis, total cellular lipids were extracted from freeze-dried cells using the Bligh-Dyer method [27], and quinones were separated and purified using a Sep-Pak plus silica column (Waters), followed by analysis using an ACUITY UPLC H-Class system (Waters). Polar lipids were extracted as previously described [28], and polar lipids analysis was perfomed using DSMZ services (Leibniz-institut DSMZ, Braunschweig, Germany).

### 2.4 Genome analysis

The genomic DNA of strain IK01^T^ was extracted using a Genomic tip 20 G (QIAGEN), followed by a quality check, removal of small molecules using a Short Read Eliminator (PacBio), and fragmentation using g-TUBE (Covaris). The library was prepared using the SMRTbell gDNA Sample Amplification Kit (PacBio) and SMRTbell Express Template Prep Kit ver.2.0 (PacBio) according to the manufacturer's instructions. Sequencing was performed using the Sequel IIe platform (PacBio) by Bioengineering Lab. Co., Ltd. (Sagamihara, Japan). Sub-reads were created from the sequences obtained by removing the overhang adaptor sequences using SMRT Link (v12.0.0.177059), and then were aligned and selected as high-fidelity (HiFi) consensus reads. The HiFi reads were assembled using Flye v2.9.1-b1780 with default parameters [29]. The draft genome sequence of strain IK01^T^ is available from GenBank/EMBL/DDBJ under the accession numbers BTFW00000000.1. Raw read sequences are available in the DDBJ under Sequence Read Archive accession number DRA016625. Annotation was performed using the DDBJ Fast Annotation and Submission Tool (DFAST v1.6.0) with default parameters [30].

## 3 Results and discussion

### 3.1 Isolation and colony characteristics

**Fig. 1:**
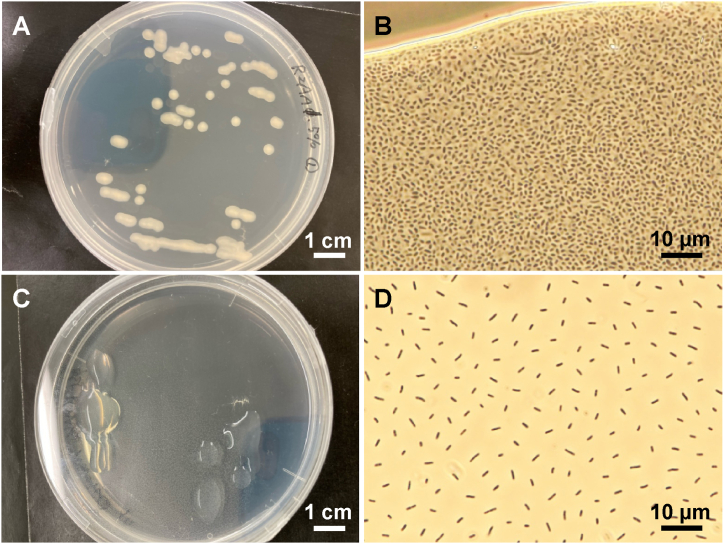
Colony morphology, cell density, and shape of strain IK01^T^. Strain IK01^T^ was grown on normal strength R2A agar (1.5 %) (A, B) and 1 % strength R2A agar (0.5 %) (C, D). The phase contrast images (B, D) show the colony samples directly taken by pipets, placed on glass slides, covered by a cover slip, and then observed under a microscope.

To isolate biofilm-forming bacteria from the MBR membrane samples, biofilm samples taken from the fouled membranes were used as inoculum samples, and cultures were conducted using several plate cultures. During the experiment, liquid droplets-like matter that could be or could not be colonies were formed on the plates. This contained microbial cells; therefore, several isolation procedures for streaking were repeated. Finally, strain IK01^T^ was isolated. The strain IK01^T^ cells were gram-negative, rod-shaped, 1–2 μm long, and 0.8–1.0 μm in width (Fig. S1), with no motility. Strain IK01^T^ formed milky-white colonies with smooth and slightly viscous surfaces on normal R2A plate medium (1.5 % agar, Fig. 1A). These characteristics of strain IK01^T^ are consistent with the description of the genus *Novosphingobium* [2]; however, the closely related species *N. capsulatum* NBRC 12533^T^ and *Novosphingobium pokkalii* L3E4^T^ have motility, whereas strain IK01^T^ does not [1,31].

At lower agar and medium concentrations, the colonies of the strain tended to become stickier, more gelatinous, almost transparent in color, and swellen (Fig. 1C, Fig. S2). The cell size was approximately twice in length that of the normal colonies (Fig. 1). This colony morphology and formation on a very low-strength medium are unique; however, these characteristics have not been previously reported in the genus *Novosphingobium*. Therefore, the relationship between the agar and medium concentrations and colony morphology was examined. The results are shown in Fig. S2. The cell distributions in normal colonies (R2A in 1.5 % agar) were crowded, whereas in the gelatinous colonies formed in 1 % R2A with 0.5 % agar, a distance was observed between the cells in low density (Fig. 1). At the very low-nutrient concentration of 1 % R2A, the closely related species *N. capsulatum* NBRC 12533^T^ did not form colonies in our experiment. Therefore, the ability of strain IK01^T^ to grow under very low nutrient conditions is a unique characteristic of this strains.

**Fig. 2:**
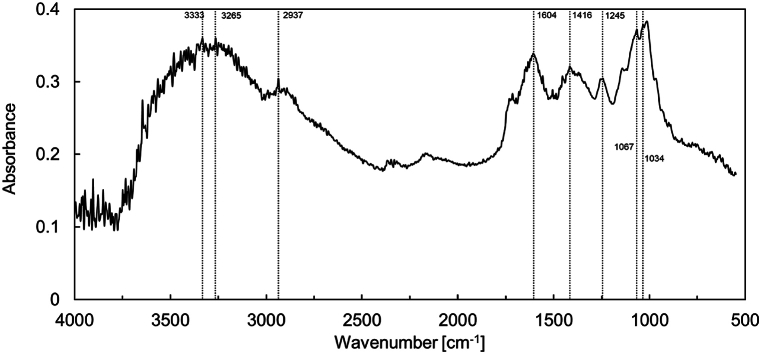
FTIR spectra of gel-like coloy fomed by strain IK01^T^.

The chemical oxygen demand (COD) concentration in 1 % strength of R2A was approximately 30 mg/L. This value was almost the same as the concentration range for the permeate of the samples [32,33]. Therefore, strain IK01^T^ can be grown in an actual environment to produce gelatinous EPS that may cause membrane fouling. Therefore, to compare the chemical characteristics of the gelatinous colonies of gel biofilms developed on the MBR membrane surface, FTIR analysis was performed (Fig. 2). Peaks assigned to polysaccharides (1014, 1067, 2937, and 3265 cm^−1^) and proteins (1245, 1416, 1604, and 3333 cm^−1^) were confirmed. These peaks were also observed in biofilm samples from the MBR membrane. This suggests that the strain IK01^T^ is one of the fouling^causing bacteria in the MBR.

### 3.2 Genotypic and phylogenetic analyses

**Fig. 3:**
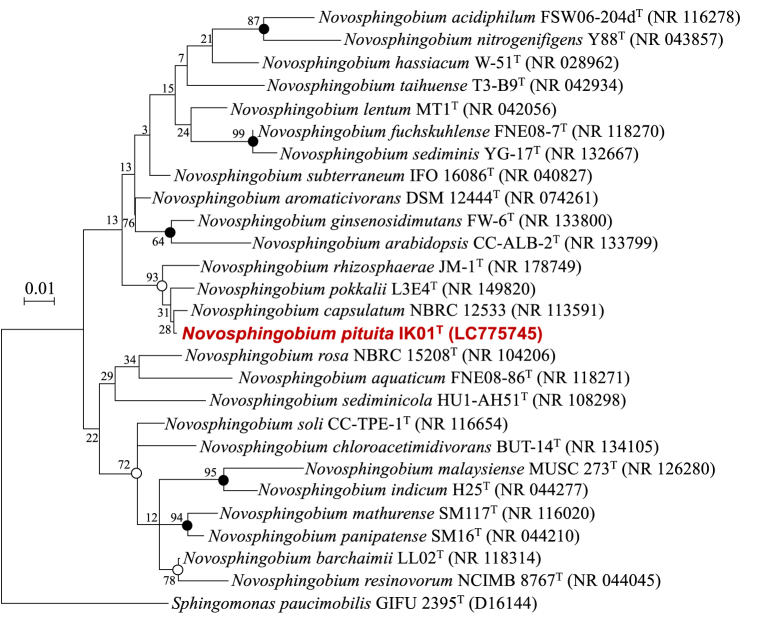
Phylogenetic position of strain IK01^T^ within the genus *Novosphingobium*. The tree was constructed based on a distance matrix analysis of the 16S rRNA gene sequence using the maximum-likelihood method. Bootstrap resampling analysis for 1000 replicates was performed for the neighbor-joining, maximum-likelihood, and maximum-likelihood methods to estimate the confidence of the tree topologies. The numbers at the nodes indicate bootstrap values for the neighbor-joining method. Branching points with support above 50 % in all three analyses are indicated by solid circles, whereas nodes with open circles indicate bootstrap support in the two analyses. Scale bar represents the 0.01 substitutions per nucleotide position.

The nearly full-length 16S rRNA gene sequence of strain IK01^T^ (1379 bp) was determined and deposited in the GenBank/EMBL/DDBJ databases under the accession number LC775745. Comparison of the similarity of strain IK01^T^ to other strains using the EzBioCloud and BenBank databases showed that it was highly similar to *N. capsulatum* NBRC 12533^T^ and *N. pokkalii* L3E4^T^ (99.19 % sequence similarity, in both). Phylogenetic analysis revealed that strain IK01^T^ formed the same clade as *N. capsulatum* NBRC 12533^T^, *N. pokkalii* L3E4^T^, and *Novosphingobium rhizosphaerae* JM-1^T^ (Fig. 3). Moreover, a phylogenomic tree based on the Genome BLAST Distance Phylogeny approach indicated that strain IK01^T^ represented a novel species-level lineage along with *N. capsulatum* NBRC 12533^T^, with *N. pokkalii* L3E4^T^ as the closest type strain (Fig. S3). These results strongly suggest that strain IK01^T^ represents a novel *Novosphingobium* species.

Four circular contigs were obtained from the draft genome sequence analysis of strain IK01^T^, and the largest genome had 65.9 × coverage, with an N50 value of 3,533,901 bp. Completeness was assessed using the CheckM version 1.2.2 [34], which showed 99.8 % completeness. The genomic DNA G + C content of strain IK01^T^ was 66.0 %, which is within the typical range (62–67 %) known for species of the genus *Novosphingobium* [2](Table 1). The assembled genome sequences, based on whole-genome sequences, average nucleotide identity (ANI), and digital DNA–DNA hybridization (dDDH) were calculated using FastANI v1.34 [35] and Genome-to-Genome Distance Calculator 3.0 [36]. The calculated ANI and dDDH values of strain IK01^T^ against the 50 reference genomes of *Novosphingobium* spp. available in the NCBI database, are shown in Table S1. Notably, *N. pokkalii* L3E4^T^ showed the highest similarity to strain IK01^T^ and showed the highest ANI and dDDH values of 83.9 % and 24.4 %, respectively.

**Table 1:** Physiological and biochemical characteristics of strain IK01^T^ and phylogenetically closely related species.

Characteristics	Strain IK01^T^	*N.capsulatum#*	*N.pokkalii*†	*N.rhizosphaerae*¶
Growth pH (optimum)	5-8 [7]	4.5–10 [7]	5-7 [7]	4.5–7.0
Growth temperature (optimum) (°C)	10-37 [[28], [29], [30], [31], [32], [33], [34], [35], [36], [37]]	10-42 [30]	18-42 [[28], [29], [30]]	15–36
NaCl range for growth (%)	0–2	0–2	0–2	nd
Motility	–	–	–	+
G + C content (mol%)	66.0	65.7	68.3	nd
**Biochemical characteristics**				
Nitrate reduction	–	+	+	±
β-galactosidase	w	+^a^	nd	+
β-glucosidase	+	+^a^	nd	+
Urease	–	-a	–	–
**Assimilation**				
Glucose	+	+	+	+
L-Arabinose	+	+	+	+
D-Mannose	–	+	nd	+
D-Mannitol	–	-a	–	–
N-Acetil-D-glucosamine	–	+^a^	nd	+
Maltose	+	+	+	+
Citric acid	–	–	–	–
DL-Malic acid	–	+	+	+
Phenyl acetate	–	-a	nd	–

-, negative; +, positive; ±, variable; w, weakly positive; nd, no data.

a Data obtained in this study. #: Data from He et al.(2022) [44], Leifson (1962) [46], and Yabuuchi et al.(1990) [1]. †: Data from Krishnan et al. (2017) [31]. ¶: Data from Kämpfer et al. (2015) [5] and Nguyen et al. (2016) (47).

**Fig. 4:**
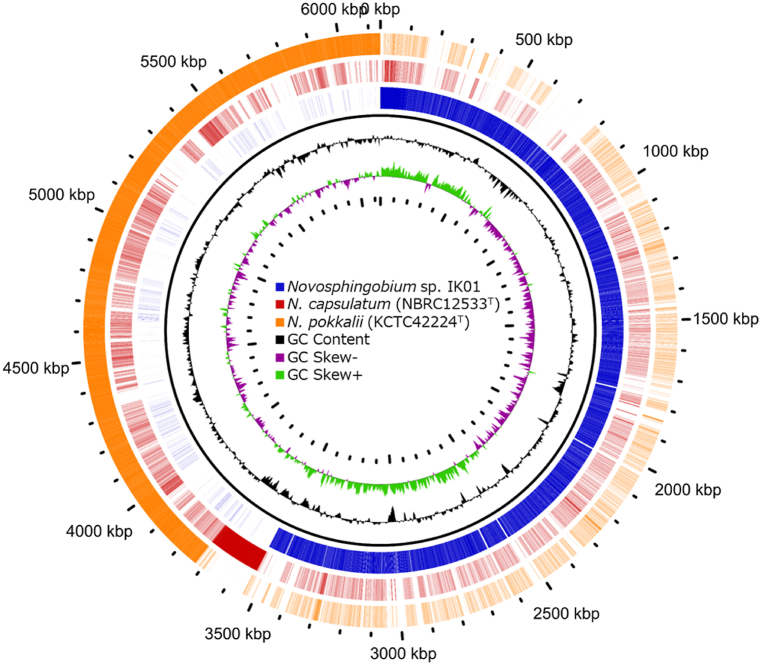
Genome sequence comparison of strain IK01^T^ and other closely related species of *N. capsulatum* NBRC12533^T^ and *N. pokkalii* L3E4^T^.

The assembled genome sequence of strain IK01^T^ contained 3449 coding sequences, 69 tRNA genes, 12 rRNA genes, and one CRISPR sequence. The GView Server [37] was used to compare the genomes of IK01^T^ with other closely related species (*N. capsulatum* NBRC12533^T^ and *N. pokkalii* L3E4^T^) (Fig. 4). Genome sequence comparisons revealed that strain IK01^T^ has unique genomic regions, including genes encoding polysaccharide biosynthesis/export proteins, membrane transport proteins, glycosyltransferases, and two-component regulatory systems. The molecular mechanisms used to produce EPS are classified into three distinct pathways: the Wzx/Wzy-dependent, ATP-binding cassette (ABC) transporter-dependent, and synthase-dependent [38,39]. The unique genes found in strain IK01^T^, *kpsE*, *kpsM*, and *kpsT*, are involved in the ABC transporter-dependent pathway, and *wza*, *uppW*, and *exoP* are involved in the Wzx/Wzy-dependent pathway [39,40]. In addition, *gmd* and *fcl*, which are responsible for the conversion of GDP-D-mannose to GDP-L-fucose, are associated with the cholanic acid production pathway, together with the fucosyl transferase WcaI [41], and were also found in strain IK01^T^. Colanic acid is a major cause of membrane fouling [42]. EnvZ and OmpR are two-component regulatory systems that govern responses to osmotic changes, and OmpR expression has been suggested to influence biofilm formation [43]. These findings suggest that the EPS of strain IK01^T^ contains fucose as a constitutive unit and that a unique glycosyltransferase and membrane transport mechanism different from those of closely related species may be involved in its production. The low-concentration medium, in which strain IK01^T^ formed gelatinous colonies, was expected to have a lower osmotic pressure than the medium with a normal concentration, suggesting that strain IK01^T^ may produce EPS in response to this low osmotic environment.

### 3.3 Chemotaxonomic and phenotypic analysis

Growth was observed at temperatures of 10–37 °C (optimum 28–37 °C), at pH 5–8, and in the presence of 0–2.0 % (w/v) NaCl. These results are consistent with those of the closely related *Novosphingobium* spp. and the genus *Novosphingobium* [44] (Table 1). The distinct feature of strain IK01^T^ is the inability of nitrate reduction and assimilation of malic acid. This strain was highly susceptible to tetracycline, penicillin G, rifampicin, and vancomycin and slightly susceptible to chloramphenicol and kanamycin. This strain was resistant to streptomycin, ampicillin, and nalidixic acid. Strain IK01^T^ exhibited β-glucosidase and β-galactosidase activities, as well as glucose, arabinose, and maltose assimilation capacities. However, nitrate reduction, mannose assimilation, N-acetyl-glucosamine assimilation, and malate assimilation, which were observed in *N. capsulatum*, were not observed in strain IK01^T^ (Table 1).

The major fatty acid of strain IK01^T^ were Summed Feature 8 (C_18:1_ *ω*7*c* and/or C_18:1_ *ω*6*c*, 57.8 %), Summed Feature 3 (C_16:1_ *ω*7*c* and/or C_16:1_ *ω*6*c*, 12.8 %), and C_14:0_ 2-OH (11.7 %). The detailed fatty acid compositions of strain IK01^T^ and a comparison with other related strains are shown in Table 2. The major fatty acid composition of strain IK01^T^ is in a range with that of closely related species. The characteristic feature of strain IK01^T^ is containing branched fatty acid of C_19:0_ cyclo ω8c. The polar lipid profile revealed phosphatidylethanolamine, two unidentified phospholipids and three aminoglycophospholipids as major compounds. Diphosphatidylglycerol, phosphatidylglycerol, and unidentified aminolipids and phospholipids were detected in lower amounts in the polar lipid profiles (Fig. S4). Quinone analysis showed that the strain IK01^T^ contained 91.3 % ubiquinone Q-10 and 8.7 % ubiquinone Q-9. These results are consistent with the previously reported fatty acid and quinone properties of *Novosphingobium* sp [45].

**Table 2:** Cellular fatty acid profiles (% of totals) of Strain IK01^T^ and phylogenetically closely related species.

Fatty acids (%)	Strain IK01^T^	*N.capsulatum*^a^	*N.pokkalii*†	*N.rhizosphaerae*^b^
**Saturated fatty acids**				
C_14:0_	1.9	0–0.4		
C_16:0_	5.2	6.5–26.8	9.6–23.5	8.5–9.8
C_17:0_	0.8			
C_18:0_	1.5	0–0.4	<1.0	1.1
C_19:0_ cyclo *ω*8*c*	6.5			
**Unsaturated fatty acids**				
C_16:1_*ω*5*c*		0.5–1.3	0.7	1.0–1.3
C_16:1_*ω*7*c*		13.1	15.7	
C_17:1_*ω*6*c*	1.8	1.8–2.3	2.1	0.9–1.2
anteiso-C17:0		1.3		
C_18:1_*ω*7*c*		47.5–68.8	49.8–67.0	66.5
C_18:1_*ω*5*c*		0.4		
**Hydroxy fatty acid**				
C_14:0_ 2-OH	11.7	11.5–15.4	10.8–11.8	12.5–13.1
**Summed feature**				
3 (C_16:1_*ω*7*c* and/or C_16:1_*ω*6*c*)	12.8	2.9–13.7	2.2	4.5
8 (C_18:1_*ω*7*c* and/or C_18:1_*ω*6*c*)	57.8	62.8		67.4

a Data from He et al.(2022) [44], Kämpfer et al. (2015) [5], Nguyen et al. (2016) [47], and Krishnan et al. (2017) [31]. †:Data from Krishnan et al. (2017) [31] and He et al.(2022) [44].

b Data from Kämpfer et al. (2015) (5), Nguyen et al. (2016) (47), and Krishnan et al. (2017) [31].

Based on the above results, we believe that the phenotypic, phylogenetic, and genomic analysis clearly indicate that strain IK01^T^ is a new species and cannot be classified as any currently published species of the genus *Novosphingobium*, although the 16S rRNA gene sequence similarity is high. In addition, strain IK01^T^ could be a fouling-causing bacterium that produces gelatinous EPS under conditions similar to the actual environment in the MBR. Based on this, we propose a novel species within the genus *Novosphingobium*, named “pituita” (Latin for “mucus").

### 3.4 Description of *Novosphingobium pituita* sp. nov

*Novosphingobium pituita* (pituita. L. fem. n. *pituita*, referring to the colony texture of type strain).

Cells are rod-shaped, 1–4 μm long, 0.8–1.0 μm wide, and non-motile. Optimal growth is observed on R2A agar at 28 °C. On R2A agar, almost gelatinous, milky-white pigmented colonies are produced after incubation for 1 d at 28 °C. On R2A agar, growth occurs between 10 °C and 37 °C, and pH values between 5 and 8. Assimilation of glucose, arabinose, and maltose is observed; however, no nitrate reduction, mannose, N-acetyl-glucosamine, or malate assimilation is observed. The fatty acid profile consisted of major amounts of summed feature 8 (C_18:1_ *ω*7*c* and/or C_18:1_ *ω*6*c*), summed feature 3 (C_16:1_ *ω*7*c* and/or C_16:1_ *ω*6*c*), and C_14:0_ 2OH. The polar lipid profile consisted of the predominant lipid phosphatidylethanolamine, two unidentified phospholipids, three aminoglycophospholipids as major compounds. Furthermore, diphosphatidylglycerol, phosphatidylglycerol, and unidentified amino lipids and phospholipids are detected in smaller amounts. The quinone system is ubiquinone Q-10. The type strain IK01^T^ (NBRC 116408^T^ = DSM 116658^T^) was isolated from a sewage treatment MBR at the Nagaoka central sewage treatment center, Nagaoka City, Niigata Prefecture, Japan (37°28′55″ N, 138°51′16″ E).

## Funding information

This research was financially supported by 10.13039/501100001691JSPS 10.13039/501100001691KAKENHI Grant Number JP19H01163 and the JST FOREST Program (Grant Number JPMJFR225T, Japan).

## Data availability

The GenBank/EMBL/DDBJ accession numbers for the 16S rRNA gene sequence and draft genome sequence of strain IK01T are LC775745 and BTFW00000000.1, respectively. Raw read sequences are available in the DDBJ under the Sequence Read Archive accession number DRA016625.

## CRediT authorship contribution statement

**Tomoya Ikarashi:** Visualization, Investigation, Formal analysis, Data curation. **Uchini S. Bandaranayake:** Writing – original draft, Investigation. **Takahiro Watari:** Project administration, Conceptualization. **Takashi Yamaguchi:** Supervision, Project administration. **Masashi Hatamoto:** Writing – review & editing, Validation, Supervision, Conceptualization.

## Declaration of competing interest

The authors declare that they have no known competing financial interests or personal relationships that could have appeared to influence the work reported in this paper.
