# Supplementary material for: Unique gel-like colony forming bacterium Novosphingobium pituita sp. nov., isolated from a membrane bioreactor (MBR) treating sewage
Source: Heliyon. 2024 Oct 1;10(19):e38795. doi: 10.1016/j.heliyon.2024.e38795 (PMC11665390; doi:10.1016/j.heliyon.2024.e38795)
Supplement: Multimedia component 1 [file mmc1.pdf]

Supplementary materials

Unique gel-like colony forming bacterium *Novosphingobium pituita* sp. nov., isolated from a membrane bioreactor (MBR) treating sewage

Tomoya Ikarashi[1], Uchini S. Bandaranayake[1], Takahiro Watari[1], Takashi Yamaguchi[1,2], Masashi Hatamoto[1]*

1 Department of Civil and Environmental Engineering, Nagaoka University of Technology, Niigata 940-2188, Japan

2 Department of Science of Technology Innovation, Nagaoka University of Technology, Niigata 940-2188, Japan

* Corresponding author

Department of Civil and Environmental Engineering, Nagaoka University of Technology, 1603-1 Kamitomioka, Nagaoka, Niigata 940-2188, Japan

Tel: +81-258-47-9637, Fax: +81-258-47-9637

E-mail address: hatamoto@vos.nagaokaut.ac.jp (Masashi Hatamoto)

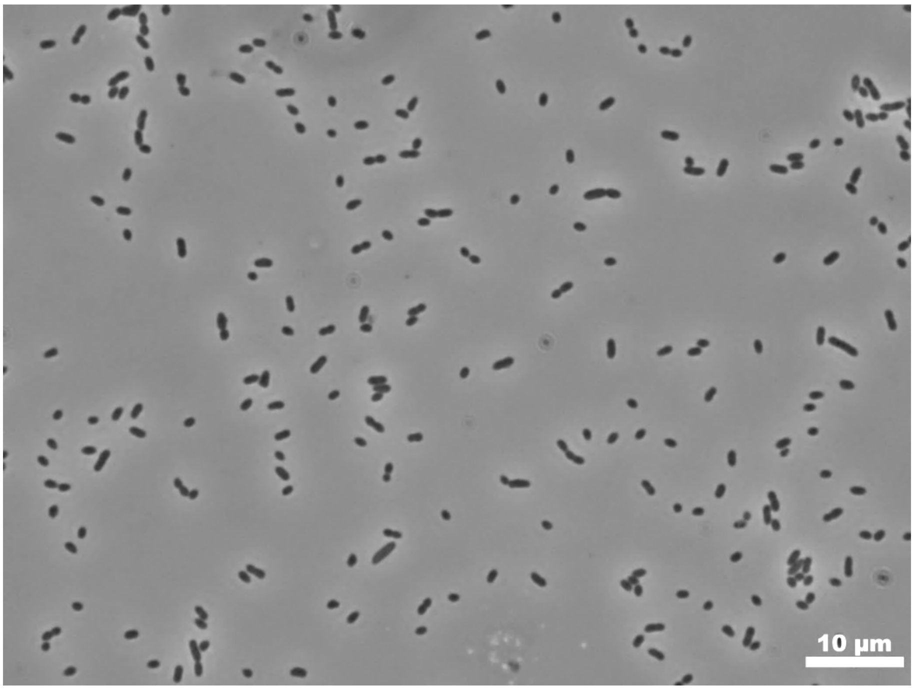

Fig.S1 Light microscope (OLYMPUS, BX53) image of strain IK01^{T} aerobically grown on R2A at 28°C overnight.

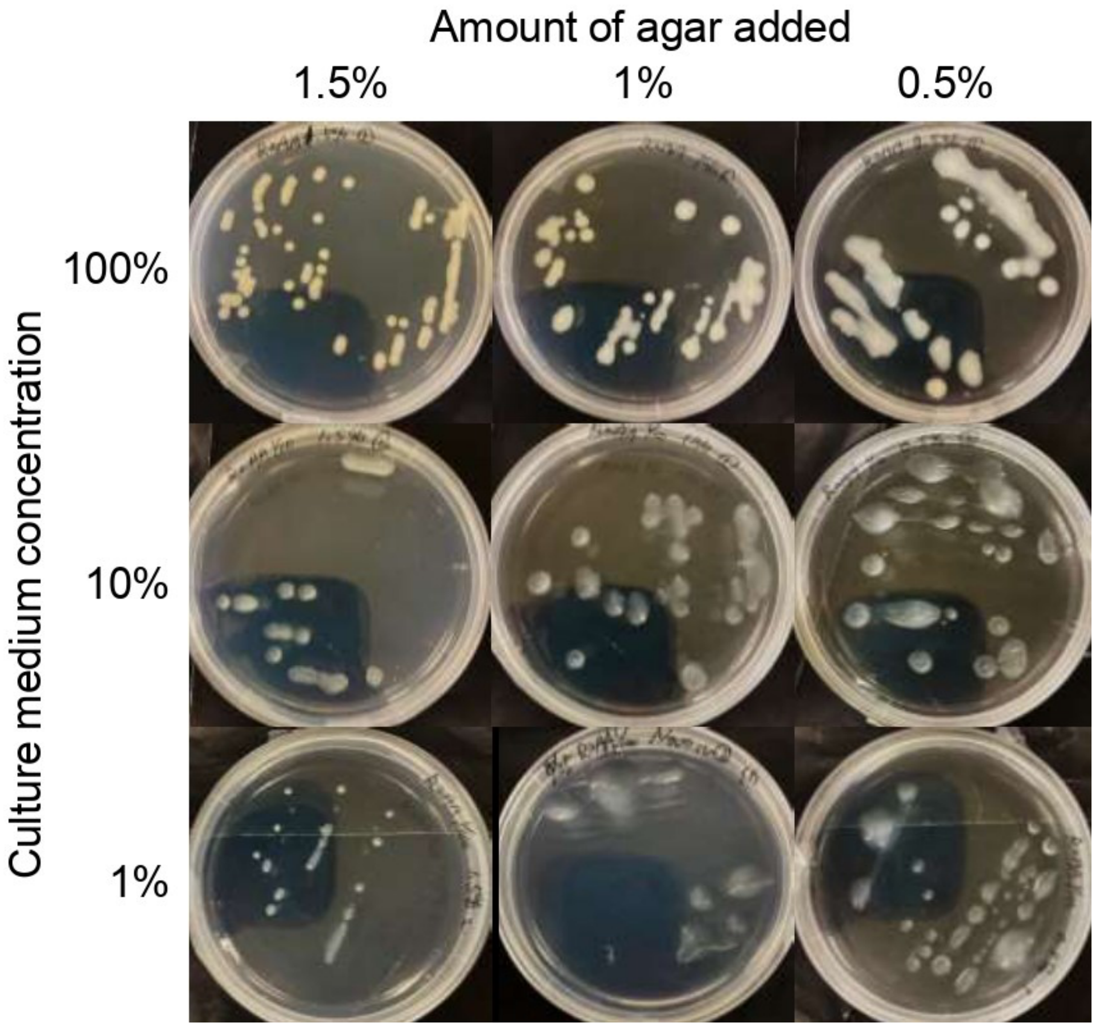

Fig.S2 The colony morphologies of strain IK01^T were investigated by culturing the strain on varying concentrations of R2A medium and agar. The concentrations of the medium were 1%, 10%, and 100%, and 0.5%, 1.0%, and 1.5% of agar were added to each medium (all concentrations in w/v %) and incubated at 28 °C for 1 week.

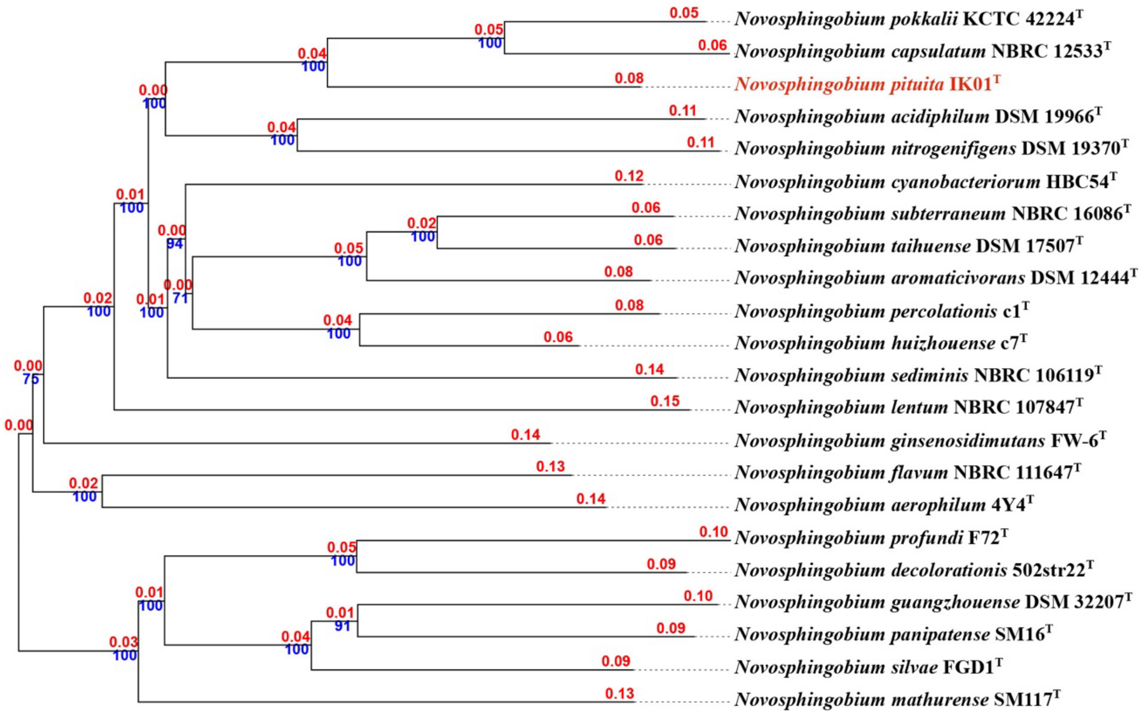

Fig. S3 Phylogenetic tree based on genome sequences showing the relationship between strain IK01^{T} and closely rated type strains. The branch lengths are scaled in terms of the GBDP distance formula d_5, and the values are indicated at each branch in the red text. Branch support was inferred from 100 pseudo-bootstrap replicates, each indicated in blue text. The tree was rooted at its midpoint.

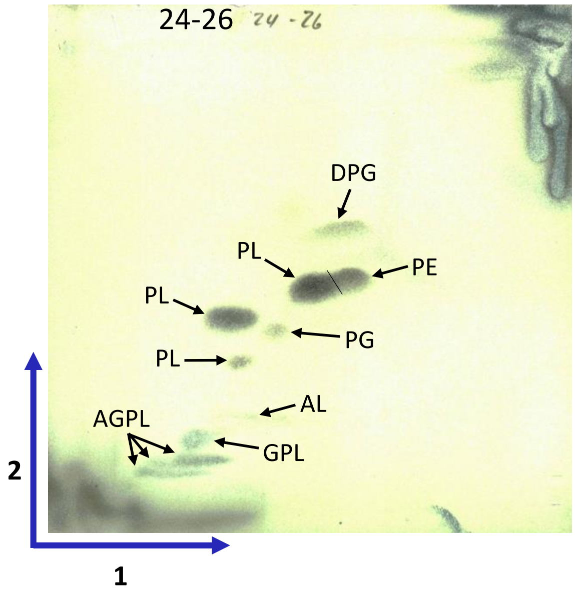

Fig.S4 Two-dimensional thin-layer chromatogram (2-D TLC) representing the total polar lipid profile of strain IK01^T. DPG, Diphosphatidylglycerol; PE, Phosphatidylethanolamine; PG, Phosphatidylglycerol; AGPL, Aminoglycophospholipid; GPL, Glycophospholipid; AL, Aminolipid; PL, Phospholipid.

Table S1 Pairwise average nucleotide identity (ANI) and digital DNA–DNA hybridization (dDDH) values (%) among strain IK01^{T} and reference strains.

Reference	ANI value	dDDH value
N. pokkalii KCTC_42224	83.9	24.4
N. capsulatum NBRC_12533	83.4	24.1
N. percolationis c1	81.3	21.7
N. huizhouense c7	81.1	22.0
N. olei TW-4	80.7	21.7
N. jiangmenense 1Y9A	80.7	21.8
N. aromaticivorans DSM_12444	80.6	21.8
N. nitrogenifigens DSM_19370	80.5	21.8
N. cyanobacteriorum HBC54	80.5	21.6
N. taihuense CGMCC_1.3432	80.1	21.2
N. hassiacum DSM_14552	80.0	20.8
N. flavum NBRC_111647	80.0	21.0
N. clariflavum 164	80.0	21.0
N. fuchskuhlense FNE08-7	80.0	20.7
N. aerophilum 4Y9	79.9	21.6
N. arvoryzae KCTC_32422	79.9	21.1
N. piscinae KCTC_42194	79.9	21.3
N. sediminis NBRC_106119	79.8	20.9
N. meiothermophilum SYSU_G00007	79.8	21.0
N. lentum NBRC_107847	79.8	20.4
N. naphthalenivorans NBRC_102051	79.8	20.9
N. lindaniclasticum LE124	79.7	21.5
N. beihaiensis B2638	79.6	20.9
N. resinovorum SA1	79.6	21.0
N. decolorationis 502str22	79.6	20.7
N. organovorum B1949	79.5	20.7

N. aureum YJ-S2-02	79.5	20.7
N. pentaromativorans US6-1	79.5	21.4
N. profundi F72	79.4	20.7
N. guangzhouense SA925	79.4	21.2
N. indicum CGMCC_1.6784	79.4	21.6
N. malaysiense MUSC_273	79.4	21.1
N. colocasiae KCTC_32255	79.4	20.8
N. mathurense SM117	79.3	20.8
N. humi KACC_19094	79.2	20.5
N. barchaimii LL02	79.2	20.9
N. ovatum FSY-8	79.2	20.7
N. acidiphilum DSM_19966	79.2	20.4
N. endophyticum CGMCC_1.15095	79.2	20.9
N. sediminicola DSM_27057	79.2	20.1
N. silvae FGD1	79.2	21.1
N. ginsenosidimutans FW-6	79.1	20.7
N. chloroacetimidivorans S00245	78.9	21.4
N. aquimarinum M24A2M	78.9	20.4
N. terrae GeG2	78.9	20.4
N. panipatense SM16	78.8	21.9
N. rosa NBRC_15208	78.7	20.4
N. fluoreni DSM_27568	78.6	20.5
N. umbonatum FSY-9	78.6	21.5
N. marinum CGMCC_1.12918	78.5	20.8
